# Large-Scale Forensic Surveillance of Seized E-Liquids Reveals an Emerging Etomidate-Analog-Centered Vaping Trend in Eastern Taiwan

**DOI:** 10.3390/toxics14070604

**Published:** 2026-07-10

**Authors:** Huei-Ru Lin

**Affiliations:** 1 Department of Laboratory Medicine and Biotechnology, Tzu Chi University, Hualien 97004, Taiwan; rita0107@gms.tcu.edu.tw; Tel.: +886-3-8565301 (ext. 12346); 2 Center for Drugs of Abuse Analysis, Tzu Chi University, Hualien 97004, Taiwan

**Keywords:** electronic cigarette, e-liquid, etomidate, new psychoactive substances (NPS), polydrug use, forensic toxicology, drug surveillance

## Abstract

The growing use of e-cigarettes as vehicles for illicit drug delivery has created an urgent challenge for forensic surveillance and public health in East Asia. This study analyzed 496 e-liquid samples seized by law-enforcement agencies in Eastern Taiwan (January–July 2025) using a full-scan GC–MS workflow validated in accordance with ANSI/ASB forensic toxicology standards. Overall, 80.8% of samples (n = 401) tested positive for psychoactive substances, with 35.4% (n = 142) containing polydrug mixtures. Etomidate dominated detections (87.0% of positive cases), followed by isopropoxate (25.4%), ketamine (9.7%), metomidate (8.5%), propoxate (7.2%), and methamphetamine (7.2%); cannabinoids (Δ^9^-THC and Δ^8^-THC) were comparatively rare (2.0% each). Predominant polydrug profiles included sedative–analog, sedative–dissociative, and sedative–stimulant combinations of acute toxicological concern. The present Eastern Taiwan dataset revealed a distinct etomidate-analog-centered adulterant profile, in marked contrast to the cannabinoid-dominated patterns reported in published seizure datasets from North America, Europe, and the Middle East. To our knowledge, seized-e-liquid surveillance data of this scale have not previously been reported for Eastern Taiwan. These findings highlight the value of seized-material toxicology as an early-warning component of forensic surveillance and support adaptive, class-level regulatory responses to the rapidly evolving landscape of designer-anesthetic vaping.

## 1. Introduction

Electronic cigarettes (e-cigarettes) have rapidly evolved from smoking-cessation tools into versatile platforms for administering psychoactive substances. Beyond nicotine and flavoring agents, chemical analyses of seized e-liquids and cartridges in Europe, North America, and the Middle East have revealed a wide spectrum of controlled substances, including cannabinoids, synthetic cannabinoid receptor agonists (SCRAs), amphetamine-type stimulants, synthetic cathinones, ketamine, and opioids. In many Western reports, illicit products are dominated by high-THC formulations and SCRAs, with associated additives such as vitamin E acetate implicated in vaping-related lung injury (EVALI) in North America [1,2,3,4]. These findings underscore the importance of systematic forensic monitoring of vaping products, which often bear misleading or incorrect labels regarding their active constituents.

Recently, attention has shifted toward e-cigarettes as carriers for sedative–hypnotic agents, particularly the intravenous anesthetic etomidate and its structural analogs. Forensic casework from East and Southeast Asia has documented etomidate, metomidate, and isopropoxate in seized e-liquids, with some studies employing rapid thermal desorption–electrospray ionization tandem mass spectrometry or probe-based high-resolution mass spectrometry to screen for these compounds in confiscated vape fluids [5]. Clinical reports from East Asia have documented severe electrolyte disturbances, adrenal insufficiency, and neuropsychiatric toxicity in users of etomidate-containing e-cigarettes [6,7,8]. These effects are attributable in part to etomidate-induced inhibition of adrenal 11β-hydroxylase [9,10], leading to life-threatening hypokalemia and secondary cardiac arrhythmia. International monitoring bodies have noted increasing detections of etomidate and its analogues in illicit drug markets globally, with East and Southeast Asia identified as a primary region of concern [11,12]. While etomidate has been increasingly flagged across multiple regions including North America, Europe, and Oceania, its emergence as a primary e-liquid adulterant at population scale remains largely confined to East and Southeast Asia [11]. An independent review by the UK Advisory Council on the Misuse of Drugs (ACMD) similarly noted that etomidate has been increasingly detected in liquids formulated for vaping, with reports from Asia significantly increasing over the last two to three years, and that some of this increase has followed the control of propofol in affected countries [13].

Against this global backdrop, data from Taiwan remain scarce despite growing concern among law-enforcement and public-health authorities. Anecdotal reports and isolated case investigations indicate that e-cigarettes have been used locally to conceal and distribute THC, ketamine, amphetamine-type stimulants, and more recently etomidate-based formulations, particularly in youth and nightlife settings. This data gap is further underscored by a February 2026 independent review by the UK ACMD, which identified East and Southeast Asia as a primary region of concern but was unable to draw on any large-scale, systematically validated seized e-liquid surveillance data from Taiwan [13]. Government scheduling decisions between 2024 and 2025, under which etomidate, metomidate, isopropoxate, and later propoxate were rapidly upgraded to higher narcotic categories, further imply a substantial and evolving misuse of imidazole-derived anesthetics in the Taiwanese illicit market [14,15,16]. However, to our knowledge, no previously published seized e-liquid surveillance study has characterized the prevalence of etomidate-analog dominance or polydrug combination patterns in Eastern Taiwan at this scale. Addressing this gap requires not only systematic analytical validation under internationally recognized criteria, but also region-specific data capable of informing both clinical awareness and regulatory policy.

The present study addresses this gap by applying a full-scan gas chromatography–mass spectrometry (GC–MS) workflow, validated in accordance with American National Standards Institute (ANSI)/AAFS Standards Board (ASB) Standards for forensic toxicology, to 496 e-liquid samples seized by law-enforcement agencies in Eastern Taiwan between January and July 2025. Using an approach that combines a routine target list, library-assisted non-target screening, and confirmation with certified reference standards, we aimed to: (i) determine the prevalence of illicit and psychoactive substances in seized e-cigarette liquids; (ii) characterize single-component versus polydrug formulations, with particular emphasis on etomidate and its analogs; and (iii) contextualize local findings within international trends in vape-based drug abuse. By providing region-specific surveillance data generated through a standards-based validation workflow, this work seeks to inform forensic practice, clinical awareness, and regulatory policy in response to the emerging threat of “designer anesthetic vaping,” while demonstrating the value of proactive, standards-based surveillance as an early-warning mechanism for emerging drug trends.

## 2. Materials and Methods

### 2.1. Chemicals and Reagents

Certified reference standards were obtained from Cerilliant Corp. (Round Rock, TX, USA) and Cayman Chemical (Ann Arbor, MI, USA). To ensure comprehensive identification, the standards were categorized into the following classes: (1) imidazole-based anesthetics: etomidate, metomidate, isopropoxate, and propoxate; (2) stimulants (Amphetamine-type and Cathinones): methamphetamine, amphetamine, 3,4-methylenedioxymethamphetamine (MDMA), 4-methylmethcathinone (mephedrone), methcathinone, cathinone, ephedrine, pseudoephedrine, phentermine, phenylpropanolamine, 3,4-methylenedioxy-N-methylcathinone (methylone) and alpha-pyrrolidinoisohexanophenone (alpha-PiHP); (3) dissociatives: ketamine and its metabolites (norketamine, dehydronorketamine); (4) cannabinoids: delta 9-tetrahydrocannabinol (Δ^9^-THC), delta 8-tetrahydrocannabinol (Δ^8^-THC), cannabidiol (CBD), cannabichromene (CBC), abnormal cannabidiol, cannabicyclol (CBL), and cannabitriol (CBT); (5) opioids: heroin, morphine, codeine, and hydrocodone; and (6) sedative-hypnotics (Benzodiazepines and Z-drugs): flunitrazepam, nimetazepam, and zolpidem.

Methanol and ethyl acetate were purchased from Mallinckrodt (Paris, KY, USA), and trifluoroacetic anhydride (TFAA) was obtained from Fluka (Buchs, Switzerland). All solvents and chemicals used in this study were of analytical or reagent grade.

### 2.2. Sample Origin and Ethical Considerations

All 496 e-cigarette liquid samples analyzed in this study were physical evidence seized by law-enforcement agencies in Eastern Taiwan (Yilan, Hualien, and Taitung Counties) between January and July 2025, and were submitted to the forensic toxicology laboratory for routine qualitative identification of controlled substances under standard chain-of-custody procedures. Each submission corresponded to an individual law-enforcement case file. However, the possibility that multiple seized units from the same incident or individual were submitted as separate cases cannot be entirely excluded, which may affect the independence of case-level counts. No human subjects, biological specimens, or directly identifiable personal information were involved. This study constituted a retrospective analysis of existing forensic case records and seized physical evidence, with no prospective human subjects research conducted. Ethical review was conducted by the Research Ethics Committee of Hualien Tzu Chi Hospital, Buddhist Tzu Chi Medical Foundation (REC No.: IRB115-030-C, approved 13 March 2026), which confirmed exemption from full human subjects review and waived the requirement for informed consent in accordance with applicable institutional regulations. Analyses were performed at a forensic toxicology laboratory certified by the Taiwan Food and Drug Administration (TFDA) for the qualitative identification of controlled substances in seized materials. The study period (January–July 2025) was selected to coincide with the completion of a fully validated identification system for all four imidazole-based anesthetic analogs (etomidate, metomidate, isopropoxate, and propoxate) at the certifying laboratory. Although etomidate prevalence had been observed since early 2024, certified reference standards for isopropoxate and propoxate did not become available until late 2024, after which a complete and internationally validated identification framework for all four analogs was established. The submitted samples represented case-based seized materials rather than epidemiological sampling units; therefore, the results should be interpreted as forensic seizure patterns rather than population prevalence of use.

### 2.3. Sample Preparation

Sample preparation followed a two-track approach based on analyte properties. Briefly, liquid samples were diluted with methanol and subjected to sonication to facilitate dissolution. An aliquot was then processed under acidic conditions and evaporated to dryness under a gentle nitrogen stream. The resulting residue was either reconstituted directly in ethyl acetate for underivatized analysis, or derivatized with trifluoroacetic anhydride (TFAA) and reconstituted in ethyl acetate to improve chromatographic resolution of selected target analytes. A 1 μL aliquot was injected into the GC–MS system for analysis.

### 2.4. Instrumentation and Analytical Conditions

Analyses were performed on an Agilent 7890A gas chromatograph coupled with a 5975C mass spectrometric detector (Agilent Technologies, Santa Clara, CA, USA), operated as a quadrupole mass spectrometer in electron ionization mode at 70 eV, equipped with a 5% phenyl-polysiloxane capillary column, with helium as carrier gas and full-scan acquisition covered a mass range sufficient to resolve all target analytes. Retention times and characteristic diagnostic ions for all routine target analytes are summarized in Table S1. The workflow was fully validated in accordance with ANSI/ASB Standards 036, 098, and 113 [17,18,19].

### 2.5. Qualitative Identification Criteria (ANSI/ASB Compliance)

Qualitative identification followed a tiered workflow. GC–MS data were first screened against the laboratory’s validated routine target list by comparing retention times and diagnostic ion ratios. Unmatched peaks of toxicological relevance were subjected to library search (NIST [20], the Cayman Chemical Spectral Library [21], and the SWGDRUG Mass Spectral Library [22], supplemented by an in-house curated reference library) and subsequently confirmed by re-analysis alongside a certified reference standard before inclusion in the reporting panel.

All identifications were performed in accordance with ANSI/ASTM E2329-25 [23], which prescribes minimum requirements for the identification of chemical substances in suspected seized drug evidence, and with ANSI/ASB Standards 113, 098, and 036 governing identification criteria, mass spectral data acceptance, and method validation, respectively [17,18,19]. Under Standard 113, a minimum of four identification points was required per analyte: one chromatographic point (retention time within the validated acceptance criteria of a concurrently analyzed reference standard) and three mass spectral points (at least three co-eluting diagnostic ions in full-scan mode) [17]. Ion abundance ratios were evaluated against reference spectra in accordance with Standard 098, with tolerances of ±20% for ions with relative intensity of 20–50% of the base peak and ±50% for ions with relative intensity less than 10% [18]. Method validation under Standard 036 encompassed selectivity, carryover, LOD, and precision at the LOD level [19]. LOD was determined by serial dilution of each target analyte, with triplicate injections at each concentration level. The lowest concentration at which all three replicates satisfied the identification criteria specified in Standard 113, namely retention time within the validated acceptance tolerance and diagnostic ion ratios within the tolerances specified in Standard 098, was defined as the LOD. Precision was expressed as the proportion of these replicate injections meeting the identification criteria at the LOD level, reflecting the qualitative nature of this workflow rather than a quantitative measure of signal variability. Carryover was evaluated by injecting a solvent blank immediately after a high-concentration standard injection and confirming the absence of diagnostic ions meeting the Standard 113 identification criteria in the blank.

### 2.6. Statistical Analysis

Regional and monthly variation in detection outcomes were evaluated using chi-square tests of independence. Regional comparisons examined detection outcome (negative, single-component positive, or multi-component positive) across the three counties (Yilan, Hualien, Taitung), using the case totals in Supplementary Table S2. Monthly comparisons examined positive versus negative detection status by month for etomidate and isopropoxate, using the monthly case totals and detection counts presented in Section 3.3. All tests were performed using IBM SPSS Statistics for Windows, Version 29.0.0.0 (IBM Corp., Armonk, NY, USA). A two-tailed *p*-value below 0.05 was considered statistically significant.

## 3. Results and Discussion

### 3.1. Method Performance and Qualitative Identification

The GC–MS method demonstrated adequate selectivity and sensitivity for qualitative identification of all target analytes (Table S1; Figure S1). No matrix interferences from common e-liquid components were observed at the retention times of the target analytes. The LOD was established at 2 μg/mL for all target analytes, with 100% precision at the decision point and no detectable carryover. All positive identifications adhered to ANSI/ASB Standards 098 and 113 criteria, with retention times and diagnostic ion ratios confirmed against concurrently analyzed certified reference standards.

This workflow was designed strictly for qualitative identification. No quantitative calibration was applied, and all results therefore represent detection frequency rather than analyte concentrations. Definitive identification required the availability of certified reference standards. Accordingly, novel analogs lacking corresponding reference standards could not be confirmed, which may have led to an underestimation of the full diversity of adulterants present in the seized samples. This limitation was partially mitigated by the library-assisted screening described in Section 2.5, although substances absent from both the routine target list and available spectral libraries remained undetectable by this workflow. Expanding non-target screening capability through the high-resolution and ambient ionization approaches discussed in Section 3.5 would further reduce this gap.

### 3.2. Chromatographic and Mass Spectral Differentiation of Etomidate and Its Structural Analogues

**Figure 1:**
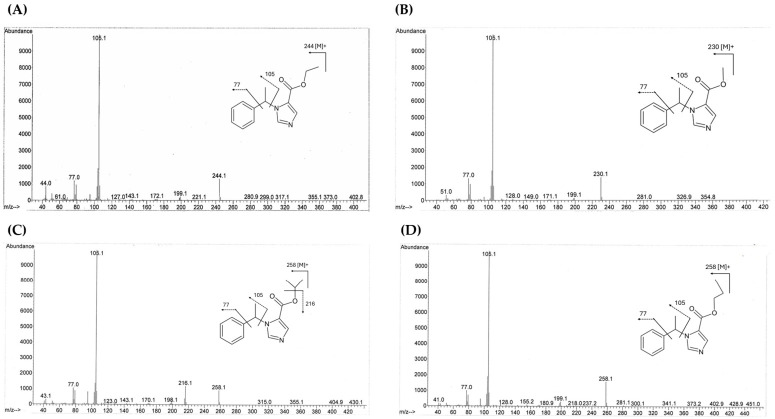
Representative electron ionization (EI) mass spectra and chemical structures of the four imidazole-based anesthetic analogs detected in this study: (**A**) etomidate, (**B**) metomidate, (**C**) isopropoxate, and (**D**) propoxate. All four compounds share a common base peak at *m*/*z* 105 (ethylimidazole cation) and a characteristic fragment at *m*/*z* 77 (phenyl cation), reflecting their common imidazole carboxylate core structure. Analog-specific molecular ions are observed at *m*/*z* 244 (etomidate), *m*/*z* 230 (metomidate), and *m*/*z* 258 (isopropoxate and propoxate); the shared molecular ion of the latter two reflects their structural isomer relationship, with chromatographic resolution providing the basis for their discrimination. For isopropoxate, the diagnostic ion at *m*/*z* 216 arises via a McLafferty-type rearrangement with neutral loss of propene (42 Da). Despite near-identical fragmentation profiles, all four analogs were chromatographically resolved (retention times summarized in Table S1), enabling unambiguous identification in both single-component and polydrug samples.

A critical challenge in this study was the differentiation of etomidate from its structural analogs—metomidate, isopropoxate, and propoxate—due to their high degree of spectral similarity. As illustrated by the annotated structures and EI mass spectra in Figure 1, these imidazole-based carboxylates share a common core structure, leading to nearly identical fragmentation patterns, with all four compounds exhibiting a dominant base peak at *m*/*z* 105 (ethyl-imidazole cation) and a significant fragment at *m*/*z* 77 (phenyl cation). To further elucidate the fragmentation pathway of isopropoxate, computational validation was performed using the NIST MS Interpreter, which indicated that the characteristic product ion at *m*/*z* 216 (C_12_H_12_N_2_O_2_^+^) arises via a McLafferty-type rearrangement: γ-hydrogen migration to the carbonyl oxygen with concomitant C–C bond cleavage and neutral loss of propene (C_3_H_6_, 42 Da), yielding a resonance-stabilized acylium/imide radical cation. Despite this spectral overlap, all four analogs were well-resolved chromatographically (Table S1), ensuring unambiguous discrimination of each compound, including in polydrug mixtures containing multiple co-occurring analytes.

### 3.3. Analysis of Seized E-Cigarette Liquid Samples

A total of 496 e-cigarette liquid samples seized in Eastern Taiwan were analyzed. The results revealed a high detection frequency of psychoactive substances, with 80.8% (n = 401) of the samples testing positive for at least one psychoactive compound (Table 1). Among the positive cases, the majority (64.6%) contained a single detected substance, while a significant proportion (35.4%, n = 142) were identified as multi-component mixtures, indicating a complex adulteration landscape in the local illicit market.

Monthly case submissions and their analyte-specific breakdown are presented in Table 2. Case numbers were lowest in January (n = 19), rose in February (n = 84), and peaked in April (n = 122). These fluctuations are attributable to operational factors: the annual renewal of service contracts delayed submission of some January cases to February, and a targeted law-enforcement operation conducted in April generated a temporary surge of seizures. Whether these case-number variations reflect any concurrent changes in underlying drug use cannot be determined from the present data. Analyte-specific detection frequencies also varied significantly across the study period. Etomidate detection frequency rose from 47.4% in January to a peak of 84.7% in June, remaining elevated at 76.6% in July (χ^2^ = 34.25, df = 6, *p* < 0.001), whereas isopropoxate detection frequency declined from 57.9% in January to 5.6% in June, remaining low at 6.3% in July (χ^2^ = 63.23, df = 6, *p* < 0.001). These opposing monthly patterns are reported descriptively rather than causally. Potential underlying drivers, including analog substitution, shifts in local supply, and regulatory scheduling effects, cannot be distinguished without longitudinal data extending beyond the scope of this cross-sectional study. Regional distribution showed that submissions originated predominantly from Hualien (n = 237, 47.8%) and Yilan (n = 213, 42.9%) Counties, with Taitung accounting for the remainder (n = 46, 9.3%). Region-specific detection patterns are summarized in Supplementary Table S2. A chi-square test of independence indicated that detection outcome (negative, single-component positive, or multi-component positive) differed significantly across counties (χ^2^ = 13.44, df = 4, *p* = 0.009), with a comparatively lower positivity rate observed in Hualien relative to Yilan.

Table 3 summarizes the detection frequencies of specific analytes. Etomidate was the most predominant substance, detected in 87.0% (n = 349) of all positive cases, followed by isopropoxate (25.4%), ketamine (9.7%), metomidate (8.5%), and propoxate (7.2%). Traditional drugs of abuse such as methamphetamine (7.2%) and cannabinoids (Δ^9^-THC and Δ^8^-THC, each 2.0%) were present but comparatively infrequent relative to the imidazole-based anesthetics. Heroin and cannabidiol (CBD) were detected at trace levels (0.5% and 0.2%, respectively). The relatively low detection rate of cannabinoids contrasts sharply with published surveillance data from North America, Europe, and the Middle East. This pattern may reflect regional supply chain differences or other market factors, and whether the regulatory scheduling of etomidate analogs contributed to this difference cannot be determined from the present cross-sectional data.

Polydrug patterns are further characterized in Table 4. The most frequently observed co-occurrence was etomidate with isopropoxate (n = 39, 9.7%), followed by etomidate with ketamine (n = 25, 6.2%) and etomidate with methamphetamine or propoxate (n = 10, 2.5%). Mixtures combining sedative–hypnotic agents with dissociatives or stimulants may produce unpredictable clinical effects and elevate the risk of acute toxicity, particularly in uncontrolled dosing contexts. Such combinations may mask early signs of intoxication while exacerbating cardiovascular strain and neuropsychiatric impairment. This concern is amplified by the known toxicological profile of etomidate itself, with clinical reports from East Asia documenting severe hypokalemia, adrenal insufficiency, and neuropsychiatric toxicity in users of etomidate-containing e-cigarettes, even in cases of presumed single-agent use [6,7,8]. In particular, the etomidate–methamphetamine combination poses a heightened toxicological risk. The stimulant effects of methamphetamine may transiently mask etomidate-induced sedation, potentially delaying recognition of physiological compromise such as hypokalemia and adrenal insufficiency, with secondary risk of cardiac arrhythmia. These etomidate-related effects are documented in clinical reports of etomidate-containing e-cigarette use [6,7].

### 3.4. Regional Profile and Comparison with International Trends

This regional contrast extends to specific patterns of adulterant co-occurrence, with etomidate frequently detected alongside its analogs isopropoxate and metomidate. While etomidate has been increasingly flagged in North America, Europe, and Oceania since 2023, its use as a primary e-liquid adulterant at scale remains concentrated in East and Southeast Asia [1,2,3,11,24].

This pattern aligns more closely with emerging reports from East and Southeast Asia describing etomidate and its structural analogs in seized e-liquids and related products. Rapid screening methods have detected etomidate, metomidate, and isopropoxate in confiscated e-liquids from mainland China and Hong Kong [5], while clinical reports have linked etomidate-containing e-cigarettes to acute toxicity including severe hypokalemia and adrenal insufficiency [6,7,8]. International monitoring bodies have likewise noted increasing detections of etomidate and its analogues on illicit drug markets globally, with the highest concentration of cases reported in East and Southeast Asia [11]. This regional concentration parallels Taiwan’s sequential scheduling of the four analogs and the ACMD’s identification of East and Southeast Asia as the primary region of concern, both introduced earlier [13,14,15,16].

In direct comparison with published seizure datasets, Almazrouei et al. analyzed 188 seized e-cigarette samples from Dubai and found that THC dominated positive cases (98%), with non-cannabinoid substances confined to just 4 of 159 positive samples and etomidate absent entirely [2]. Li et al., by contrast, described etomidate analogs in a more limited seizure dataset but did not document dominant market prevalence [5]. This study documents etomidate-analog dominance in seized e-cigarette liquids from Eastern Taiwan using a GC–MS workflow validated against ANSI/ASB forensic toxicology standards. To our knowledge, no comparable published dataset currently exists for this region.

From a public health and regulatory perspective, the sequential scheduling of etomidate, metomidate, isopropoxate, and propoxate between 2024 and 2025 illustrates the inherent challenge of compound-by-compound reactive regulation in addressing a rapidly diversifying class of designer anesthetics, and underscores the need for proactive, class-level monitoring frameworks. Proactive forensic surveillance programs, such as the one reported here, are essential to provide early warning of emerging analogs and to support evidence-based, class-level regulatory responses. Unlike biological toxicology specimens, which reflect drug exposure after use has occurred, seized e-liquid surveillance can identify emerging adulterant formulations before widespread clinical harm is recognized, offering a prospective dimension to forensic public health intelligence.

### 3.5. Limitations and Future Directions

This study has several limitations that should be considered when interpreting the findings. First, the dataset comprises 496 e-liquid samples seized by law-enforcement agencies in Eastern Taiwan over a seven-month period, beginning immediately after certified reference standards for all four imidazole-based anesthetic analogs became available and a complete identification system was established. Consequently, the study period does not capture the earlier emergence of etomidate observed since early 2024, prior to the availability of reference standards for its structural analogs. The dataset may not be representative of vaping products circulating in other regions of Taiwan or in non-seized consumer markets. Whether the adulteration patterns observed here reflect broader national trends, or are specific to the supply chain characteristics of this geographically distinct area, cannot be determined from the present data alone. Furthermore, the composition of the seized sample set is inevitably influenced by law-enforcement priorities and operational capacity, which may introduce a sampling bias distinct from the actual distribution of adulterated products in circulation. Detection frequencies therefore reflect the profile of materials encountered by law-enforcement rather than an unbiased estimate of market prevalence. Second, as detailed in Section 3.1, the strictly qualitative nature of the workflow and its dependence on certified reference standards limit both dose-related interpretation and the detection of novel analogs lacking corresponding standards. Finally, the study did not include biological specimens or clinical outcome data, and the toxicological implications of specific polydrug compositions must therefore be inferred from existing literature rather than directly correlated with patient presentations.

Future work should expand surveillance to additional regions of Taiwan and extend the observation period to capture temporal trends in etomidate-analog use and possible displacement by newly emerging substances. Integrating high-resolution mass spectrometry or ambient ionization techniques, such as TD-ESI/MS or probe-ESI/QTOF, could improve non-target screening capacity and facilitate earlier recognition of novel analogs [5,25]. Combining seized-sample analysis with clinical or roadside toxicology data would help link specific e-liquid compositions to real-world intoxication outcomes, including the cardiovascular, neuropsychiatric, and electrolyte disturbances associated with etomidate-containing products. From a forensic toxicology perspective, the present findings highlight the need for adaptive target lists and inter-laboratory data sharing frameworks capable of responding to the rapid emergence of designer anesthetic analogs, a challenge that extends beyond Eastern Taiwan to forensic laboratories across East and Southeast Asia. Collaborative spectral databases sharing validated reference data, and case metadata across the region would support faster detection and more timely regulatory responses.

## 4. Conclusions

This study documents etomidate-analog dominance in seized e-cigarette liquids from Eastern Taiwan using a validated GC–MS surveillance workflow. To our knowledge, no comparable dataset of this scale has previously been reported from this region. Among 496 seized samples, 80.8% tested positive for at least one psychoactive substance, with etomidate detected in 87.0% of positive cases, a pattern markedly distinct from the cannabinoid-centered adulteration commonly reported in published seizure datasets from North America, Europe, and the Middle East. The presence of polydrug mixtures combining sedative, dissociative, and stimulant agents presents significant acute toxicological risks, particularly given the documented clinical harms associated with etomidate-induced adrenocortical suppression. The sequential scheduling of etomidate, metomidate, isopropoxate, and propoxate between 2024 and 2025 illustrates the inherent challenge of compound-by-compound reactive regulation in addressing a rapidly diversifying class of designer anesthetics, and underscores the need for proactive, class-level monitoring frameworks. The present findings further suggest that forensic laboratory networks in East and Southeast Asia would benefit from harmonized reporting protocols and inter-laboratory spectral data sharing, enabling earlier recognition of emerging designer-anesthetic analogs across jurisdictions.

## Figures and Tables

**Table 1:** Summary of seized e-cigarette liquid cases and GC–MS detection outcomes in Eastern Taiwan (January–July 2025).

Category	n	% of All Cases
Total e-cigarette liquid cases analyzed	496	100.0
Negative for targeted analytes	95	19.2
Positive for at least one targeted or confirmed psychoactive substance	401	80.8
Single-component positive cases	259	64.6 *
Multi-component positive cases	142	35.4 *

* Percentages calculated among all positive cases (n = 401).

**Table 2:** Monthly distribution of detected analytes in seized e-liquid samples.

Month	January	February	March	April	May	June	July	Total
Total seized e-liquid samples analyzed	19	84	62	122	73	72	64	496
Etomidate	9	46	34	95	55	61	49	349
Isopropoxate	11	31	23	21	8	4	4	102
Ketamine	1	10	2	13	4	2	7	39
Metomidate	2	6	5	5	4	9	3	34
Propoxate	1	10	1	6	0	8	3	29
Methamphetamine	0	6	2	7	6	2	6	29
Δ^9^-THC	0	7	0	1	0	0	0	8
Δ^8^-THC	0	7	0	1	0	0	0	8
Heroin	0	0	0	0	0	0	2	2
CBD	0	0	0	1	0	0	0	1

**Table 3:** Detection frequency and prevalence of individual psychoactive substances in seized e-liquid samples.

Substance	Detections (n)	Prevalence Among Positive Cases (%) ^a^	Prevalence Among All Cases (%) ^a^
Etomidate	349	87.0	70.4
Isopropoxate	102	25.4	20.6
Ketamine	39	9.7	7.9
Metomidate	34	8.5	6.9
Propoxate	29	7.2	5.8
Methamphetamine	29	7.2	5.8
Δ^9^-THC	8	2.0	1.6
Δ^8^-THC	8	2.0	1.6
Heroin	2	0.5	0.4
Cannabidiol (CBD)	1	0.2	0.2

^a^ Detections are analyte-level counts; individual samples may contain more than one analyte. Percentages are calculated relative to positive cases (n = 401) and all seized cases analyzed (n = 496), respectively, and do not sum to 100%.

**Table 4:** Prevalence and ranking of the six most frequent polydrug combinations identified in seized e-cigarette liquid samples.

Rank ^a^	Combination Profile (Detected Substances)	Count (n)	Prevalence Among Positive Cases (%) ^b^	Prevalence Among All Cases (%) ^b^	Classification
1	Etomidate, Isopropoxate	39	9.7	7.9	Multi-analog imidazole–anesthetic combination
2	Etomidate, Ketamine	25	6.2	5.0	Imidazole anesthetic + dissociative combination
3	Etomidate, Methamphetamine	10	2.5	2.0	Imidazole anesthetic + stimulant combination
3	Etomidate, Propoxate	10	2.5	2.0	Multi-analog imidazole–anesthetic combination
5	Etomidate, Isopropoxate, Methamphetamine	7	1.7	1.4	Multi-analog imidazole–anesthetic combination
5	Etomidate, Isopropoxate, Metomidate	7	1.7	1.4	Multi-analog imidazole–anesthetic combination

^a^ Standard competition ranking was applied: items with equal values receive the same rank, and the next rank number is skipped accordingly. ^b^ Percentages were calculated relative to all positive cases (n = 401) and all seized cases analyzed (n = 496), respectively. Because polydrug combinations are not mutually exclusive, percentages do not sum to 100%. Note: The six combinations listed account for 98 of the 142 multi-component positive cases (69.0%). The remaining 44 cases (31.0%) involved less frequent combinations that are not individually listed.

## Data Availability

The original contributions presented in this study are included in the article/Supplementary Materials. Further inquiries can be directed to the corresponding author.

## Supplementary Materials

The following supporting information can be downloaded at: https://www.mdpi.com/article/10.3390/toxics14070604/s1, Table S1. Retention times and monitored ions used for GC–MS qualitative identification of the laboratory’s routine target analytes. Table S2. Regional distribution by detection pattern. Figure S1. Total ion chromatography of GC/MS analysis of routine target analytes.

## References

[1] Krishnasamy V.P., Hallowell B.D., Ko J.Y., Board A., Hartnett K.P., Salvatore P.P., Danielson M., Kite-Powell A., Twentyman E., Kim L. (2020). Update: Characteristics of a Nationwide Outbreak of E-Cigarette, or Vaping, Product Use-Associated Lung Injury—United States, August 2019–January 2020. Morb. Mortal. Wkly. Rep..

[2] Almazrouei E.S., Bintamim A.A., Khalil S.E.A., Alremeithi R., Gewily S. (2022). The Identification of Drugs of Abuse in E-Cigarette Samples Seized in Dubai between 2016 and 2020. Forensic Sci. Int..

[3] Duffy B., Li L., Lu S., Durocher L., Dittmar M., Delaney-Baldwin E., Panawennage D., LeMaster D., Navarette K., Spink D. (2020). Analysis of Cannabinoid-Containing Fluids in Illicit Vaping Cartridges Recovered from Pulmonary Injury Patients: Identification of Vitamin E Acetate as a Major Diluent. Toxics.

[4] Timmerman A., Lyphout C., Verougstraete N., Coopman V., Stove C. (2026). Fast and Reliable in Vitro Activity-Based Detection of Synthetic Cannabinoid Receptor Agonists in E-Liquids. Arch. Toxicol..

[5] Li M., Lin B., Zhu B. (2024). Rapid Screening of Etomidate and Its Analogs in Seized E-Liquids Using Thermal Desorption Electrospray Ionization Coupled with Triple Quadrupole Mass Spectrometry. Toxics.

[6] Wu W., Xia C., Gan L., Liao S., Yan Y. (2024). Etomidate-Induced Hypokalemia in Electronic Cigarette Users: Two Case Reports and Literature Review. Front. Endocrinol..

[7] Chung Y.K., Cheung Y.T., Chan C.S.Y., Wong C.C., Fu A.C.C., Lam Y.Y., Lee C.Y. (2025). Adrenal Insufficiency Due to Etomidate Inhalation via Electronic Cigarettes: Three Local Cases. Hong Kong Med. J..

[8] Ng C.Z., Al-Aamari H.H.S., Low L.T.K., Zhang M.W. (2025). The Emerging Landscape of Etomidate E-Cigarettes Use. Addiction.

[9] Wagner R.L., White P.F., Kan P.B., Rosenthal M.H., Feldman D. (1984). Inhibition of Adrenal Steroidogenesis by the Anesthetic Etomidate. N. Engl. J. Med..

[10] de Jong F.H., Mallios C., Jansen C., Scheck P.A., Lamberts S.W. (1984). Etomidate Suppresses Adrenocortical Function by Inhibition of 11β-Hydroxylation. J. Clin. Endocrinol. Metab..

[11] United Nations Office on Drugs and Crime (UNODC) Increasing Detections of Etomidate and Analogues on Illicit Drug Markets Is Becoming a Global Concern. https://www.unodc.org/LSS/Announcement/Details/8774c132-4b30-477c-9ceb-46ce384223fd.

[12] United Nations Office on Drugs and Crime (UNODC) (2025). Synthetic Drugs in East and Southeast Asia: Latest Developments and Challenges.

[13] Advisory Council on the Misuse of Drugs (ACMD) ACMD Review of the Evidence on the Use and Harms of Etomidate. https://www.gov.uk/government/publications/acmd-review-of-the-evidence-on-the-use-and-harms-of-etomidate/acmd-review-of-the-evidence-on-the-use-and-harms-of-etomidate-accessible.

[14] Executive Yuan (Taiwan) (2024). Announcement of the Amendment to the Schedule of Narcotic Drugs: Listing of Etomidate, Metomidate, and Isopropoxate as Category 3 Narcotics.

[15] Executive Yuan (Taiwan) (2024). Announcement of the Amendment to the Schedule of Narcotic Drugs: Upgrading Etomidate, Metomidate, and Isopropoxate to Category 2 Narcotics.

[16] Executive Yuan (Taiwan) (2025). Announcement of the Amendment to the Schedule of Narcotic Drugs: Listing of Propoxate as Category 2 Narcotics.

[17] (2024). Standard for Identification Criteria in Forensic Toxicology.

[18] (2023). Standard for Mass Spectral Analysis in Forensic Toxicology.

[19] (2019). Standard Practices for Method Validation in Forensic Toxicology.

[20] National Institute of Standards and Technology (NIST) (2017). NIST/EPA/NIH Mass Spectral Library.

[21] Cayman Chemical Company (2024). Cayman Chemical Spectral Library.

[22] Scientific Working Group for the Analysis of Seized Drugs (SWGDRUG) (2023). SWGDRUG Mass Spectral Library.

[23] (2025). Standard Practice for Identification of Seized Drugs.

[24] Cozier G.E., Gardner M., Craft S., Skumlien M., Spicer J., Andrews R., Power A., Haines T., Bowman R., Manley A.E. (2025). Synthetic Cannabinoids in E-Cigarettes Seized from English Schools. Addiction.

[25] Lin M., Zhang Z., He Q., Hao H., Xiang P., Zhao J. (2025). Rapid Determination of Etomidate and Its Structural Analogues in E-Liquid by Probe Electrospray Ionization Quadrupole Time-of-Flight Mass Spectrometry. J. Pharm. Biomed. Anal..

