# Supplementary material for: Large-Scale Forensic Surveillance of Seized E-Liquids Reveals an Emerging Etomidate-Analog-Centered Vaping Trend in Eastern Taiwan
Source: Toxics. 2026 Jul 10;14(7):604. doi: 10.3390/toxics14070604 (PMC13419080; doi:10.3390/toxics14070604)
Supplement: Supplementary file 1 [file toxics-14-00604-s001.zip › toxics-4418633-supplementary.pdf]

Table S1. Retention times and monitored ions used for GC–MS qualitative identification of the laboratory’s routine target analytes.

Analyte	RT (min)	Monitored ions (*m/z*)
Methamphetamine-TFAA	3.60	154, 118, 110
4-methylmethcathinone-TFAA	4.11	119, 154, 91
MDMA-TFAA	5.68	162, 289, 135, 110, 154
alpha-PiHP-TFAA	5.78	140, 98, 141
Etomidate	6.38	105,104, 244, 77
Metomidate	6.56	105,104, 230, 77
Isopropoxate	6.67	105, 104, 216, 258, 77
Ketamine-TFAA	6.79	270, 262, 236, 110, 298, 152, 125, 276
Propoxate	7.09	105, 104, 258, 77
Δ9-THC-TFAA	7.93	410, 367,395, 339, 297, 327, 313
Flunitrazepam-TFAA	9.6	312, 285, 294, 266, 313, 286
Heroin	11.1	369, 327, 310, 268, 215, 204
Nimetazepam-TFAA	11.5	294, 295, 267, 268, 248, 220

Analytes not included in the routine target list but reported in the study (Δ8-THC and CBD) were identified through library-assisted screening and confirmed by re-analysis with certified reference standards, including retention-time and EI mass-spectral agreement.

Table S2. Regional distribution by detection pattern.

	Region		
	**Yilan**	**Hualien**	**Taitung**
Total E-cigarette related Specimens number	213	237	46
Negative for targeted analytes	27	61	7
single-component positive cases	121	111	27
Multi-component positive cases	65	65	12

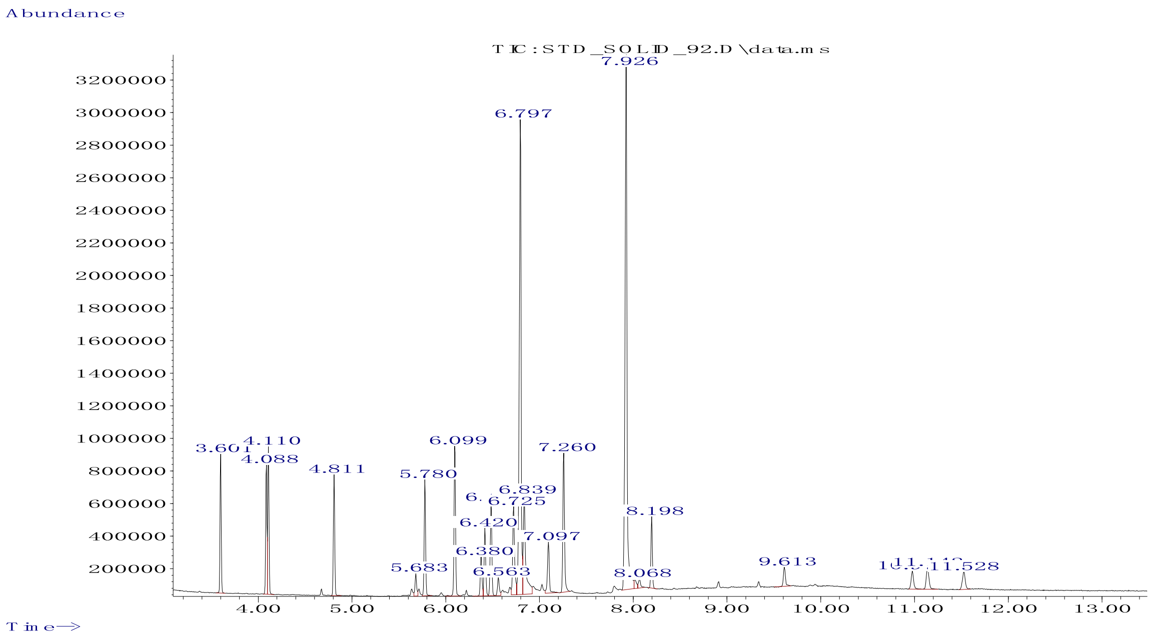

Figure S1. Total ion chromatography of GC/MS analysis of routine target analytes.
